# Supplementary material for: The Systems Biology Research Tool: evolvable open-source software
Source: BMC Syst Biol. 2008 Jun 29;2:55. doi: 10.1186/1752-0509-2-55 (PMC2446383; doi:10.1186/1752-0509-2-55)
Supplement: Additional file 1 — SBRT Archive. An archive of the current version of the Systems Biology Research Tool. [file 1752-0509-2-55-S1.zip › sbrt-1.4.0/doc/developers_guide/api/sbrt/shell/mng/MapManagerInput.html]

MapManagerInput


|  |  |  |
| --- | --- | --- |
| **Overview** \| **Package** \| **Class** \| **Use** \| **Tree** \| **Deprecated** \| **Index** \| **Help** | |  |
| **PREV CLASS**   **NEXT CLASS** | **FRAMES**    **NO FRAMES**     **All Classes** |  |
| SUMMARY: NESTED \| FIELD \| CONSTR \| METHOD | DETAIL: FIELD \| CONSTR \| METHOD |  |


---


## sbrt.shell.mng Class MapManagerInput<K,V>

```
java.lang.Object
  sbrt.shell.mng.MapManagerInput<K,V>
```

**Type Parameters:**: `K` - the key type.: `V` - the value type.

**All Implemented Interfaces:**: ManagerInput<K,V>

---

``` public class MapManagerInput<K,V> extends java.lang.Object implements ManagerInput<K,V> ```

This class is a concrete implemenation of `ManagerInput`. It provides
a way to construct manager inputs from maps.

**Author:**
:   This class was written and documented by
    Jeremiah Wright while in the Wagner lab.

---

**Constructor Summary**

`MapManagerInput(java.util.Map<K,V> input)`
  
          Constructs a new manager input from the provided map with a
`null` name.

`MapManagerInput(java.util.Map<K,V> input,
java.lang.String name)`
  
          Constructs a new manager input from the provided map and
name.


| **Method Summary** | |
| --- | --- |
| `java.lang.String` | `getName()`             Returns the name of this manager input. |
| `V` | `getOptionalValue(java.lang.Object key)`             Returns the value corresponding to the provided key. |
| `V` | `getRequiredValue(java.lang.Object key)`             Returns the value corresponding to the provided key. |
| `boolean` | `hasValueFor(java.lang.Object key)`             Indicates if a value exists for the provided key in this manager input. |
| `java.util.Map<K,V>` | `toMap()`             Returns the information contained in this input as a map. |

**Methods inherited from class java.lang.Object**

`clone, equals, finalize, getClass, hashCode, notify, notifyAll, toString, wait, wait, wait`

**Constructor Detail**

### MapManagerInput

```
public MapManagerInput(java.util.Map<K,V> input,
                       java.lang.String name)
```

:   Constructs a new manager input from the provided map and
    name.

    **Parameters:**: `input` - keys mapped to their respective values.: `name` - the name given to this manager input.

---


### MapManagerInput

```
public MapManagerInput(java.util.Map<K,V> input)
```

:   Constructs a new manager input from the provided map with a
    `null` name.

    **Parameters:**: `input` - keys mapped to their respective values.


**Method Detail**

### hasValueFor

```
public boolean hasValueFor(java.lang.Object key)
```

:   Indicates if a value exists for the provided key
    in this manager input.

    :   **Specified by:**: `hasValueFor` in interface `ManagerInput<K,V>`
    :   **Parameters:**: `key` - the key whose presence in this input is to be tested. **Returns:**: `true` if this input contains a value for the specified key.

---


### getOptionalValue

```
public V getOptionalValue(java.lang.Object key)
```

:   Returns the value corresponding to the provided key.

    :   **Specified by:**: `getOptionalValue` in interface `ManagerInput<K,V>`
    :   **Parameters:**: `key` - the key whose value is to be returned. **Returns:**: the value corresponding to the provided key, or `null` if the value does not exist.

---


### getRequiredValue

```
public V getRequiredValue(java.lang.Object key)
```

:   Returns the value corresponding to the provided key.

    :   **Specified by:**: `getRequiredValue` in interface `ManagerInput<K,V>`
    :   **Parameters:**: `key` - the key whose value is to be returned. **Returns:**: the value corresponding to the provided key. **Throws:**: `ApplicationException` - if the value does not exist.

---


### toMap

```
public java.util.Map<K,V> toMap()
```

:   Returns the information contained in this input as a map.

    :   **Specified by:**: `toMap` in interface `ManagerInput<K,V>`
    :   **Returns:**: keys mapped to their respective values.

---


### getName

```
public java.lang.String getName()
```

:   Returns the name of this manager input.

    :   **Specified by:**: `getName` in interface `ManagerInput<K,V>`
    :   **Returns:**: the name of this manager input.


---


|  |  |  |
| --- | --- | --- |
| **Overview** \| **Package** \| **Class** \| **Use** \| **Tree** \| **Deprecated** \| **Index** \| **Help** | |  |
| **PREV CLASS**   **NEXT CLASS** | **FRAMES**    **NO FRAMES**     **All Classes** |  |
| SUMMARY: NESTED \| FIELD \| CONSTR \| METHOD | DETAIL: FIELD \| CONSTR \| METHOD |  |


---
